# Uptake in non-affected bone tissue does not differ between [^18^F]-DCFPyL and [^68^Ga]-HBED-CC PSMA PET/CT

**DOI:** 10.1371/journal.pone.0209613

**Published:** 2018-12-20

**Authors:** Jochen Hammes, Melanie Hohberg, Philipp Täger, Markus Wild, Boris Zlatopolskiy, Philipp Krapf, Bernd Neumaier, Klaus Schomäcker, Carsten Kobe, Matthias Schmidt, Markus Dietlein, Alexander Drzezga

**Affiliations:** 1 Department of Nuclear Medicine, University Hospital of Cologne, Cologne, Germany; 2 Institute of Radiochemistry and Experimental Molecular Imaging, University Hospital of Cologne, Cologne, Germany; 3 Institute of Neuroscience and Medicine–5 (Nuclear Chemistry), Research Center Jülich, Jülich, Germany; Biomedical Research Foundation, UNITED STATES

## Abstract

**Introduction:**

[^68^Ga]PSMA-HBED-CC and [^18^F]DCFPyL show a high potential for the detection of recurrent prostate cancer. While ^18^F-based tracers have several advantages in availability and image resolution, their sensitivity in the skeleton might be impaired by released [^18^F]fluoride due to its high bone affinity. In turn, chemically unbound trivalent ^68^Ga might also accumulate in osseous tissue, in cases of occupied binding sites of plasma proteins and thereby influence bone signal.

**Methods:**

A comparison of average bone SUV was performed in 17 bone-negative and 4 bone-positive patients. All patients underwent PET/CT 125 minutes after application of [^18^F]DCFPyL and 73 minutes after application of [^68^Ga]PSMA-HBED-CC at another date.

**Results:**

Native SUVs in unaffected bone tissue and SUVs relative to liver uptake were lower in [^18^F]DCFPyL (0.49) than in [^68^Ga]PSMA-HBED-CC scans (0.52). SUVs relative to gluteal muscles did not differ between the two tracers. Average lesional SUVs did not differ between tracers.

**Conclusion:**

No difference of average bone signal intensity was observed for [^18^F]DCFPyL-PET/CT in comparison to [^68^Ga]PSMA-HBED-CC scans indicating that diagnostic assessment of the skeleton is not affected by non-specific accumulation of free [^18^F]fluoride or ^68^Ga.

## Introduction

Both ^68^Ga- and ^18^F-labeled tracers for imaging of prostate-specific membrane antigen (PSMA) such as [^68^Ga]PSMA-HBED-CC and [^18^F]DCFPyL have already demonstrated high potential for the detection of recurrent prostate cancer [1–4]. While ^18^F-based tracers exhibit several advantages in terms of availability, production amount and image resolution, based on experiences with other ^18^F based tracers, unspecific high tracer retention in bone tissue not affected by metastases might be responsible for a decrease of sensitivity in these areas due to defluorination. Although it has not been reported as a major limitation of this particular tracer before, it is conceivable that [^18^F]fluoride released from [^18^F]DCFPyL could cause a higher physiological background signal in osseous structures because it is a substrate of bone metabolism [5] and bone uptake has been traditionally used by other investigators as a measure of defluorination [6–8]. In turn, free trivalent ^68^Ga that has dissolved from the chelating agent, might also have a high binding affinity to osseous structures, however this would require a state of occupied binding sites on plasma proteins or very low plasma transferrin levels [9]. In this study, we systematically compared the physiological tracer uptake in bone tissue in patients without osseous metastases who underwent both [^68^G]aPSMA-HBED-CC and [^18^F]DCFPyL PET/CT using EBONI, a software tool to automatically quantify PET tracer uptake in bone tissue, published recently by us [10].

## Methods

### Compliance with ethical standards

All procedures performed in studies involving human participants were in accordance with the ethical standards of the institutional and/or national research committee and with the 1964 Helsinki declaration and its later amendments or comparable ethical standards. This article does not contain any studies with animals performed by any of the authors. The institutional review board of the University Hospital of Cologne has approved this study. Written informed consent was obtained from all individual participants included in the study.

21 patients underwent PSMA PET/CT with both [^68^Ga]PSMA-HBED-CC and [^18^F]DCFPyL in our department in a time frame of no longer than one month. Four of these patients were diagnosed with bone metastases. Average age of patients was 66.5 (+/- 8.5) years. Repeated PET/CTs with different PSMA-tracers were performed for clinical reasons (mostly due to a negative [^68^Ga]PSMA-HBED-CC PET scan in patients with biochemical recurrence) as published previously [2, 3]. All PET/CT scans were performed on a Siemens Biograph mCT scanner (mCT 128 Flow Edge, Siemens, Knoxville, USA). PET/CT imaging was performed in accordance with the Institutional Review Board. Written informed consent was obtained from all individual participants included in the study. No change in therapeutic regimen had taken place between both scans. Acquisition and reconstruction (OSEM: 4 iterations, 12 subsets) was performed according to a protocol published previously [2]. In short, PET acquisition began 125 (+/- 12) minutes after injection of 311 (+/- 61) MBq [^18^F]DCFPyL and 73 (+/- 14) minutes after injection of 162 (+/1 54) MBq [^68^Ga]PSMA-HBED-CC. Average Radiochemical purity was well above 95% and did not differ significantly between tracers. For accurate determination of the activity of the radiopharmaceuticals the dose calibrator ISOMED 2010 (NUVIA Instruments GmbH, Dresden, Germany) was used. Quality control procedures of this class IIb medical device are carried out regularly according to DIN 6855–11.

**Fig 1:**
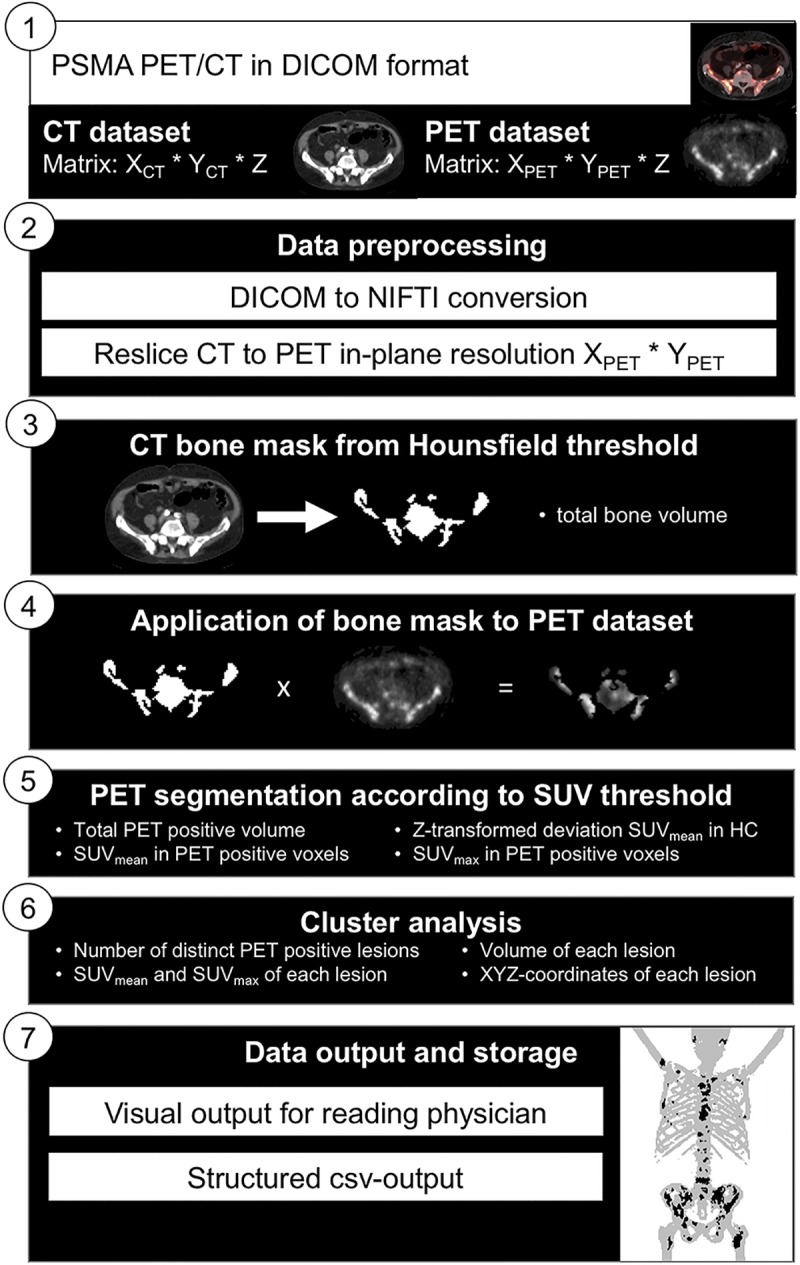
Algorithmic functionality of automated bone-VOI delineation and metastasis load quantification with EBONI in an exemplary patient with known osseous metastases of prostate cancer. csv = comma-separated values; HC = healthy controls. This figure was originally published in JNM [10]. Hammes et al. EBONI: A Tool for Automated Quantification of Bone Metastasis Load in PSMA PET/CT. J Nucl Med. 2018 Jul;59(7):1070–1075 by the Society of Nuclear Medicine and Molecular Imaging, Inc.

**Fig 2:**
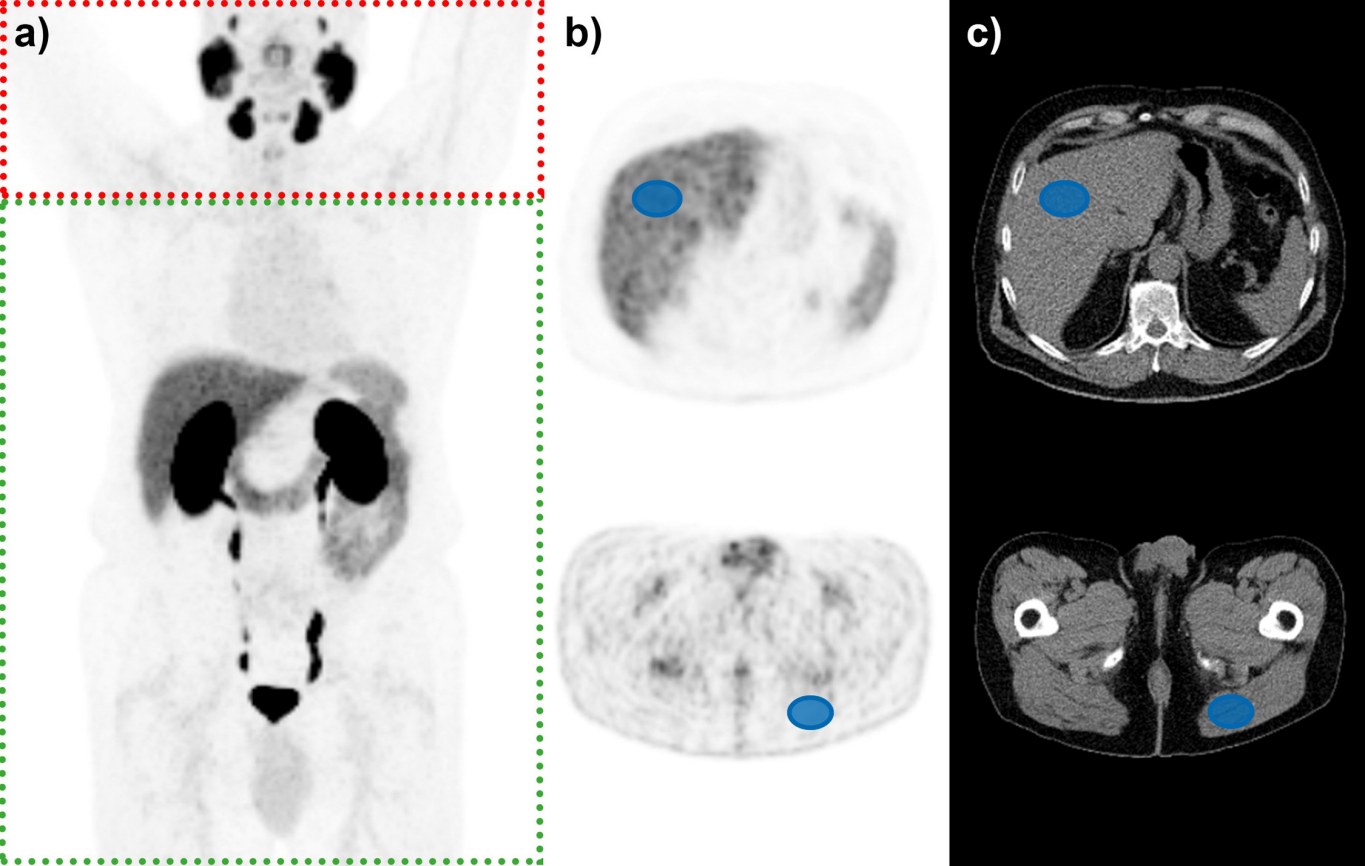
Exemplary PET dataset (a) and placement of VOIs (b,c). Red-dotted area was excluded from the analysis to avoid spillover into bone voxels caused by uptake in salivary and lacrimal glands.

The absence of bone metastases in 17 patients was confirmed by two experienced nuclear medicine physicians (PT, JH). Average standardized uptake value (SUV) in bone tissue (SUV_bone_) in the bone negative group was determined automatically with EBONI. As previously published, this software calculates tracer uptake selectively within bone tissue as defined by CT information based on Hounsfield-density [10]. The algorithmic function of EBONI is depicted in Fig 1. To avoid artefacts caused by spillover from unspecific uptake in extraosseous tissue, i.e. in facial bones due to uptake in the lacrimal and salivary glands [11], PET/CT datasets were analyzed starting from the upper thorax downwards. Average SUV in volumes of interest (VOIs) in the homogenous liver tissue (SUV_liver_) as well as in the musculus gluteus maximus (SUV_gluteus_) were extracted with VINCI (http://vinci.sf.mpg.de) [12]. VOIs were drawn manually so that they did not contain regions with elevated tracer uptake suspicious of being a metastasis. Minimum VOI volume was 5 ml. Exemplary VOIs are shown in Fig 2. Ratios between SUV_bone_ and individual SUV_Liver_ and SUV_gluteus_ were calculated. In the bone positive group, SUV_max_ and SUV_mean_ of the osseous metastases were determined by semiautomatic lesion segmentation in Siemens syngo.via (Lesion threshold 40% of SUV_max_). Results were checked for normal distribution with Kolmogorov-Smirnov test and compared with Wilcoxon signed rank test. P values below 0.05 were considered significant. Fig 1 illustrates the algorithmic functionality of the creation of the bone-VOI with EBONI. Since we wanted to analyze tracer uptake in patients with no bone metastases, the CT-derived bone-VOI was applied without an SUV-threshold in step 4.

## Results

**Fig 3:**
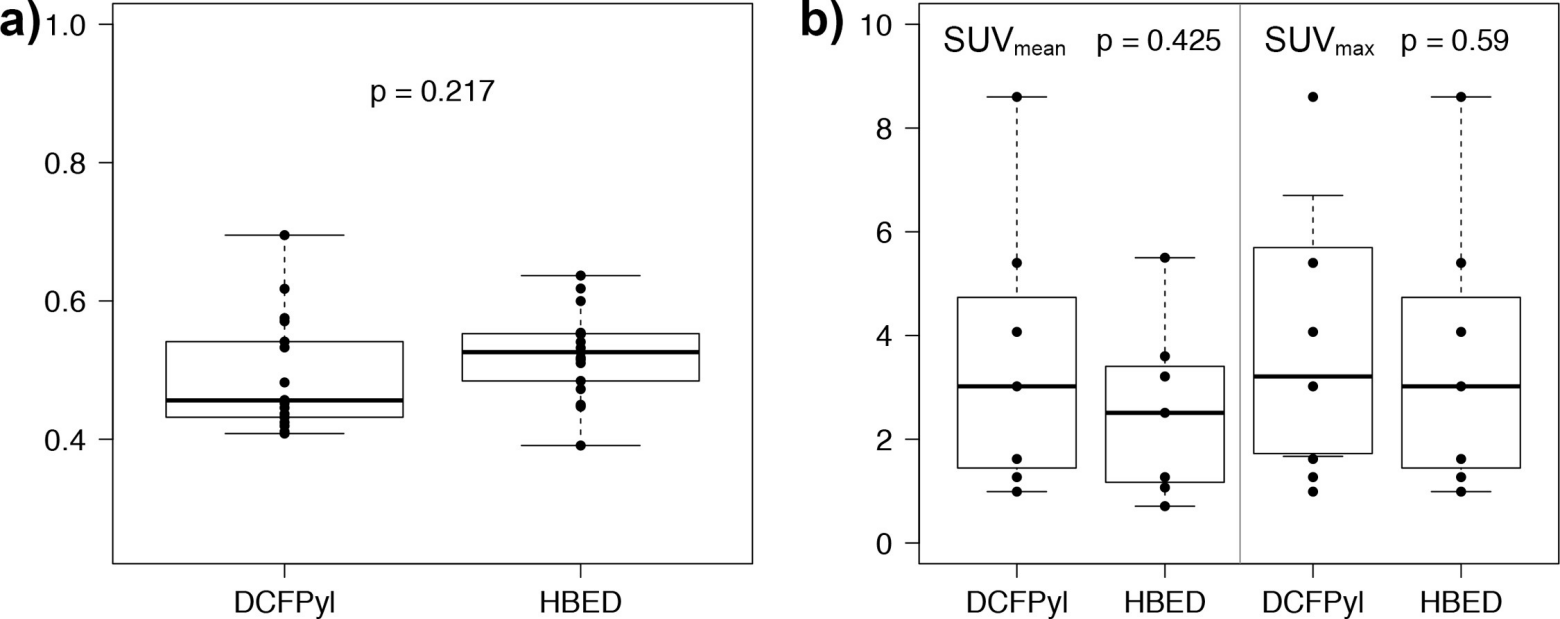
a) Average SUVs in unaffected bone tissue in 17 patients without bone metastases. b) SUV_mean_ and SUV_max_ in a total of seven bone metastases in the group of patients with bone metastases.

Quantitative results with average SUV-values in the bone negative group are listed in Table 1 and displayed in Fig 3A. SUVs were not normally distributed. Unprocessed SUV_bone_ and SUV_bone_ relative to individual SUV_liver_ was lower for [^18^F]DCFPyL scans than for [^68^Ga]PSMA-HBED-CC scans. SUV_bone_ relative to individual SUV_gluteus_ did not differ between [^18^F]DCFPyL and [^68^Ga]PSMA-HBED-CC. Radiochemical purity was 95.5% for [^18^F]DCFPyL and 98.9% for [^68^Ga]PSMA-HBED-CC but did not differ significantly (p = 0.07). Average SUV_max_ and SUV_mean_ of bone metastases in the bone positive group are listed in Table 2 and Fig 3B. Average SUVs in bone metastases and SUVs relative to the gluteal reference region were numerically higher in the [^18^F]DCFPyL scans, although the difference did not exceed the significance threshold.

**Table 1:** Quantitative results in bone negative group. Average values and standard deviation.

	^18^F-DCFPyL	^68^Ga-PSMA-HBED-CC	p (Wilcoxon)
SUV_liver_	6.1 (1.2)	4.6 (1.0)	<0.01*
SUV_gluteus_	0.35 (0.13)	0.36 (0.08)	0.62
SUV_bone_	0.49 (0.08)	0.52 (0.06)	0.03*
SUV_bone_/ SUV_liver_	0.082 (0.02)	0.117 (0.019)	<0.01*
SUV_bone_/ SUV_gluteus_	1.50 (0.37)	1.54 (0.39)	0.55

* indicates statistical significance

**Table 2:** Quantitative results in bone metastases.

	DCFpyL	HBED	p
Bone metastasis SUV_max_	4,56 (3,86)	3,57 (2,74)	0,113
SUV_mean_	3,54 (2,78)	2,55 (1,71)	0,092
SUV_liver_	6,81 (1,38)	5,38 (0,5)	0,080
SUV_gluteus_	0,35 (0,07)	0,36 (0,04)	0,869
SUV_max_/ SUV_liver_	0,64 (0,45)	0,69 (0,56)	0,588
SUV_mean_/ SUV_liver_	0,5 (0,33)	0,49 (0,35)	0,891
SUV_max_/ SUV_gluteus_	12,69 (9,44)	10,79 (9,53)	0,189
SUV_mean_/ SUV_gluteus_	9,9 (6,86)	7,62 (5,92)	0,062

## Discussion

Here, we demonstrate that average SUV of [^18^F]DCFPyL and [^68^Ga]PSMA-HBED-CC in bone tissue (unaffected by metastases of prostate cancer) is comparable between tracers. Our data leads to the conclusion, that, for these PSMA-tracers, chemically unbound radionuclides apparently only occur in quantities that are small enough not to affect the intensity of the background signal in bone tissue. To date, both tracer families are considered widely interchangeable for most clinical applications [13], while there is evidence that the average SUV of PSMA positive lesions is higher for [^18^F]DCFPyL than for [^68^Ga]PSMA-HBED-CC (when acquired according to the mentioned procotcols), as reported earlier by our group [2]. Here, we could not confirm a significant difference of lesional SUVs between the tracers, however, we attribute this fact to the small number of bone-positive patients we had available to include in this study.

It needs to be taken into account that the imaging protocols were not entirely comparable for the two tracers. Although the same scanner was used for acquisition of all images, as compared to [^68^Ga]PSMA-HBED-CC, a higher dose of [^18^F]DCFPyL has been injected and the images were acquired significantly later, which corresponds to the usual procedure for this tracer. The [^18^F]DCFPyL tracer's higher half-life and production yield enable this approach, which has been chosen because a better lesion contrast and resulting higher sensitivity can be expected [2, 3].

We believe that these differences in the acquisition protocols should not have considerably affected the results of this study. First, it appears reasonable to compare the two tracers according to the usual conditions of their use in everyday practice. Second, we would not expect that later image acquisition or injection of higher tracer doses would lead to a decrease in relative bone uptake of the tracer.

The level of skeletal binding depends on the radiochemical purity achieved during tracer production. In our sample, the radiochemical purities of both tracers did not differ significantly. This further stresses the understanding that skeletal signal is only marginally affected by unbound ^18^F-flouride and does not lead to differences in image quality. In addition to this observation, considering the typical biological distribution of free Gallium, an affection of the bone signal due to unspecific accumulation of the nuclide released from [^68^Ga]PSMA-HBED-CC could only be expected, if the Gallium binding sites of peripheral plasma proteins were occupied or plasma protein concentrations were unusually low. Therefore, under normal circumstances, an in influence of free ^68^Ga could only be expected if it was administered in amounts that are several orders of magnitude higher than those typically administered in human PET examinations [9].

The aforementioned advantages of [^18^F]DCFPyL together with the findings of this study support the notion that the ^18^F-labeled tracer [^18^F]DCFPyL may be advantageous for clinical routine application. These findings, however, cannot be generalized to other ^18^F-labeled PSMA-tracers like [^18^F]PSMA-1007 [14] with different in vivo characteristics and therefore will have to be evaluated accordingly.
